# Antenatal depressive symptoms and early initiation of breastfeeding in association with exclusive breastfeeding six weeks postpartum: a longitudinal population-based study

**DOI:** 10.1186/s12884-019-2195-9

**Published:** 2019-01-29

**Authors:** Karin Cato, Sara M. Sylvén, Marios K. Georgakis, Natasa Kollia, Christine Rubertsson, Alkistis Skalkidou

**Affiliations:** 1 Department of Women’s and Children’s Health, Uppsala University Hospital, 751 85 Uppsala, SE Sweden; 2 Department of Neuroscience, Psychiatry, Uppsala University Hospital, Uppsala, Sweden; 3 Department of Hygiene, Epidemiology and Medical Statistics, National and Kapodistrian University of Athens, Athens, Greece; 4 Department of Nutrition and Dietetics, School of Health Science and Education, Harokopio University, Athens, Greece

**Keywords:** Antenatal depression, Breastfeeding initiation, Exclusive breastfeeding, Breastfeeding discontinuation

## Abstract

**Background:**

Depressive symptoms negatively impact on breastfeeding duration, whereas early breastfeeding initiation after birth enhances the chances for a longer breastfeeding period. Our aim was to investigate the interplay between depressive symptoms during pregnancy and late initiation of the first breastfeeding session and their effect on exclusive breastfeeding at six weeks postpartum.

**Methods:**

In a longitudinal study design, web-questionnaires including demographic data, breastfeeding information and the Edinburgh Postnatal Depression Scale (EPDS) were completed by 1217 women at pregnancy weeks 17–20, 32 and/or at six weeks postpartum. A multivariable logistic regression model was fitted to estimate the effect of depressive symptoms during pregnancy and the timing of the first breastfeeding session on exclusive breastfeeding at six weeks postpartum.

**Results:**

Exclusive breastfeeding at six weeks postpartum was reported by 77% of the women. Depressive symptoms during pregnancy (EPDS> 13); (OR:1.93 [1.28–2.91]) and not accomplishing the first breastfeeding session within two hours after birth (OR: 2.61 [1.80–3.78]), were both associated with not exclusively breastfeeding at six weeks postpartum after adjusting for identified confounders. Τhe combined exposure to depressive symptoms in pregnancy and late breastfeeding initiation was associated with an almost 4-fold increased odds of not exclusive breastfeeding at six weeks postpartum.

**Conclusions:**

Women reporting depressive symptoms during pregnancy seem to be more vulnerable to the consequences of a postponed first breastfeeding session on exclusive breastfeeding duration. Consequently, women experiencing depressive symptoms may benefit from targeted breastfeeding support during the first hours after birth.

**Electronic supplementary material:**

The online version of this article (10.1186/s12884-019-2195-9) contains supplementary material, which is available to authorized users.

## Background

Breastfeeding is widely known to benefit both the baby and the mother [1, 2]. As several of the breastfeeding benefits for both mother and infant appear to be further strengthened by longer duration and exclusive breastfeeding, the World Health Organization (WHO) recommends exclusive breastfeeding during the first six months after birth [3]. The Swedish recommendations are in line with the WHO guidelines, although they include an amendment declaring that the introduction of “tiny sensations” of solid food from the age of four months is harmless if it does not affect continuous breastfeeding [4]. In general, breastfeeding rates are high among new mothers [5]. In 2014, 96% of Swedish mothers were breastfeeding one week after birth [5]. However, rates of exclusive breastfeeding are shown to be decreasing, especially towards the late postpartum period, being around 64% at two months, and plummeting to 16% at six months postpartum [5].

Several factors could affect the duration of breastfeeding. Specifically early breastfeeding initiation have been associated with longer exclusive breastfeeding duration, as well as a strengthened breastfeeding self-efficacy [6–8]. On the other hand, breastfeeding self-efficacy is not only associated with longer breastfeeding duration, but also with lower levels of depressive symptoms [9]. To enable an early breastfeeding session after birth, skin-to-skin care between mother and newborn seems to have several benefits [10]. Accordingly, recommendations suggest that breastfeeding should be initiated as soon as possible after birth [3] and the baby should be preferably placed with the mother and not separated until the first breastfeeding session takes place [11].

Depression is among the most common maternal complications during childbearing, with an estimated 20% of mothers experiencing an episode within the first three months postpartum. One of the strongest predictor for postpartum depression is the incidence of depression during pregnancy [12–14]. There is evidence demonstrating a complex interplay between perinatal depression and breastfeeding with a potentially amphi-directional association. A recent systematic review dealing with this issue concludes that depression during pregnancy predicts a shorter breastfeeding duration which may consequently increase postpartum depressive symptoms [15].

The aim of this study was to assess the interplay between antenatal depressive symptoms and early initiation of breastfeeding on exclusive breastfeeding at 6 weeks postpartum, when there is no obvious reason for introducing other foods or drinks, in a population-based cohort of Swedish pregnant women.

## Methods

### Study population

This study was undertaken as part of the BASIC (Biology, Affect, Stress, Imaging and Cognition) project, a population-based longitudinal study ongoing since September 2009, which included more than 4500 pregnancies and focuses on antenatal and postnatal maternal wellbeing; detailed information may be found elsewhere [16]. All pregnant women attending the routine ultrasound examination, in gestational week 17–20, at the Uppsala University Hospital, receive written information about the project and are invited to participate. Exclusion criteria at recruitment include inability to adequately communicate in Swedish, protected identity, pathologic pregnancies as diagnosed by the routine ultrasound or intrauterine demise and age < 18 years. Written consent was obtained from all the participating women. After obtaining consent, participating women are asked to complete self-administered web-based questionnaires at recruitment, at gestational week 32 and at six weeks postpartum. The BASIC project has a participation rate of 22% and the study protocol has been approved by the Regional Research and Ethics Committee of Uppsala (no. 2009/171). For the whole cohort, 81.3% of those giving consent to participate complete the questionnaire in gestational week 32 and for the whole cohort, 80.9% at 6 weeks postpartum.

**Fig. 1:**
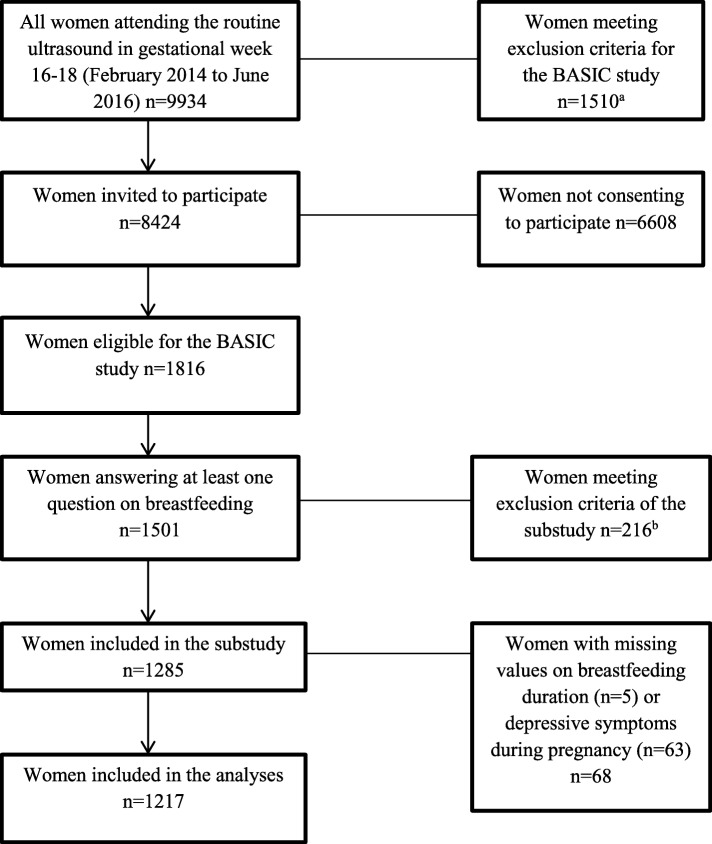
Flowchart of the selection of the study participants

The present study is a sub-study of the BASIC project, based on data collected from February, 2014, to June, 2016 (*n* = 1501 unique participants). Women, who gave birth before the 36th gestational week (*n* = 24), had missing values for gestational week at birth (*n* = 131), gave birth in another hospital (*n* = 8), did not initiate breastfeeding (*n* = 23), as well as mothers of twin pregnancies (*n* = 19) and repetitive participants due to a new pregnancy (*n* = 17) were excluded from the study. Women with missing values on breastfeeding duration (*n* = 5) or depressive symptoms during pregnancy (*n* = 63) were also excluded from this sub-study. Finally, 1217 women were included in the analyses (Fig. 1).

### Outcome measures and study variables

In the present study, the main outcome was exclusive breastfeeding at six weeks postpartum, dichotomized into exclusive breastfeeding versus partial breastfeeding or cessation of breastfeeding at six weeks postpartum, as self-reported by the participants. Several background and antenatal sociodemographic, lifestyle and medical variables, such as age (< 25, 25–34, > 35 years), body mass index (BMI) before pregnancy (< 25, 25–29, 30–34, > 35 kg/m^2^), educational level (high school or lower vs college/university), smoking during pregnancy (yes vs no), medical history of depression (no history of depression vs history of depression), employment status (working/studying vs on maternity leave/sick/unemployed) were included in the first web-questionnaire, answered by the women at gestational week 17–20. Obstetric variables such as parity (primiparas vs multiparas), mode of delivery (vaginal birth, vacuum extraction, planned caesarean section, emergency caesarean section), use of local epidural anesthesia as well as obstetric complications in pregnancy (any of the following, as self-reported or reported in medical records; vaginal bleeding during pregnancy, significant Braxton-Hicks contractions, symphysiolysis, diabetes, hypothyroidism, hypertonia and preeclampsia) and postpartum complications (hemorrhage > 1000 ml, manual placental expulsion, Apgar score at 5 min < 7, admission to the neonatal unit, laceration grade III or IV) were obtained from the medical records. The questions on timing of the first breastfeeding session after birth, experience of the first breastfeeding session (very positive/positive vs negative/very negative) and use of the hands-on approach, i.e. when health care professionals force the baby to the breast by using their hands and touching the woman’s breast and the baby in order to stimulate latch-on and breastfeeding, during the first breastfeeding session (no vs yes) were answered by the women at six weeks postpartum. Depressive symptoms during pregnancy were determined by the Swedish version of the Edinburgh Postnatal Depression Scale (EPDS) at gestational week 17–20 and/or gestational week 32. In line with previous studies, a score of > 13, giving a sensitivity of 77% and a specificity of 94%, was considered indicative of presence of depressive symptoms [17]. EPDS was also used for evaluation of depressive symptoms in the postpartum periods and particularly at six weeks postpartum; a cut-off of ≥12, giving a sensitivity of 96% and a specificity of 49%, was used, as recommended for a Swedish sample [18].

### Statistical analyses

Crude analyses were performed to assess the possible associations of the study variables with breastfeeding at six weeks postpartum. Odds Ratios (OR) and 95% confidence intervals (95% CI) were calculated through cross-tabulations. A multivariable logistic regression model was fitted to estimate the specific effect of breastfeeding initiation later than two hours after birth and depressive symptoms during pregnancy on exclusive breastfeeding at six weeks postpartum. There was no multicollinearity between the variables included in the model. In order to identify potential mediators and confounders, we created a conceptual directly acycled graph (DAG), based on literature data and available variables (Additional file 1: Figure S1). According to the DAGs, a direct effect model (model 1) was designed including age, mode of giving birth, depressive symptoms during pregnancy, educational level and parity, as well as an indirect effect model (model 2) investigating also the mediators effect, which included age, mode of giving birth, history of depression, depressive symptoms during pregnancy, educational level, parity, use of the hands-on approach during the first breastfeeding session and experience of the first breastfeeding session. The analysis was thereafter repeated stratified by the mode of giving birth.

To additionally explore the interplay between depressive symptoms during pregnancy and breastfeeding initiation later than two hours after birth, a composite variable was created and multivariate associations with the outcome variable were investigated. The composite variable included the following categories: (a) women with no depressive symptoms during pregnancy who initiated breastfeeding within two hours after birth (set as the reference category), (b) women with no depressive symptoms during pregnancy who initiated breastfeeding more than two hours after birth, (c) women with depressive symptoms during pregnancy who initiated breastfeeding within two hours after birth, (d) women with depressive symptoms during pregnancy who initiated breastfeeding > two hours after birth.

SPSS version 24 was used for the statistical analyses.

## Results

In total, 1217 women were included in the current study (Fig. 1). The mean age of the participating women was 31.4 (SD: 4.5) years and 47% of them were primiparas. Among the women who breastfed for the first time within two hours as well as the women breastfeeding for the first time after two hours, the mean age was 31.0 (SD: 4.5). Education of college or university level was reported by 80% of the participants and 92% were employed or studying. Among women, breastfeeding for the first time within 2 h after birth, the mean BMI was 24 (SD: 4.0). Among women, breastfeeding for the first time after two hours postpartum, the mean BMI was 24 (SD: 4.8). Only 7 and 1% of the women had a BMI in the spectrum 30–34.9 and ≥ 35 kg/m^2^ before pregnancy, respectively, whereas the proportion of those smoking during pregnancy was below 1 %. Seventy-nine percent of the women gave birth vaginally. Regarding the main variables of interest, the prevalence of depressive symptoms during pregnancy was 13%. Notably, 78% of the women reported initiation of breastfeeding within two hours after birth and 77% reported breastfeeding exclusively at six weeks postpartum. As presented in Table 1, the univariate analyses showed increased odds of delayed (> 2 h) breastfeeding initiation with primiparity, BMI ≥35, planned or emergency caesarean section and vacuum extraction, use of local epidural anesthesia during labour and postpartum obstetric complications. Furthermore, initiation of breastfeeding > two hours after birth was associated with a negative experience of the first breastfeeding session and experience of the hands-on approach during the first breastfeeding session.

**Table 1:** Distribution of study variables by breastfeeding or not within two hours after delivery

	Breastfeeding within two hours after delivery		
	Yes (*n* = 953)	No (*n* = 264)	
	*n* (%)	*n* (%)	OR (95% CI)^a^
Background variables			
Age (years)			
*< 25*	52 (6)	16 (6)	1.01 (0.54–1.89)
*25–34*	677 (71)	180 (68)	0.88 (0.64–1.20)
*≥ 35*	224 (24)	68 (26)	*Reference*
Parity			
*Primiparas*	398 (45)	179 (73)	3.24 (2.38–4.42)
*Multiparas*	483 (55)	67 (27)	*Reference*
Educational level			
*College/University*	745 (81)	209 (81)	*Reference*
*High school or lower*	181 (19)	49 (19)	0.97 (0.68–1.37)
Employment status			
*Unemployed*	71 (8)	26 (10)	1.36 (0.85–2.19)
*Employed/Student*	857 (92)	230 (90)	*Reference*
Pre-pregnancy BMI^b^ (kg/m^2^)			
* < 25*	667 (72)	177 (69)	*Reference*
*25–29.9*	188 (20)	62 (24)	1.24 (0.89–1.73)
*30–34.9*	64 (7)	4 (1)	0.24 (0.09–0.66)
* ≥ 35*	12 (1)	15 (6)	4.7 (2.17–10.2)
History of depression			
*Yes*	276 (30)	80 (32)	1.06 (0.79–1.44)
*No*	638 (70)	174 (68)	*Reference*
Pregnancy and Birth variables			
Smoking at 17th week of pregnancy			
*Yes*	7 (0.8)	1 (0.4)	1.88 (0.23–15.4)
*No*	838 (99.2)	225 (99.6)	*Reference*
Depressive symptoms during pregnancy^c^			
*Yes*	117 (12)	37 (14)	1.17 (0.78–1.73)
*No*	836 (88)	227 (86)	*Reference*
Pregnancy complications^d^			
*Yes*	501 (53)	153 (59)	1.24 (0.94–1.64)
*No*	439 (47)	108 (41)	*Reference*
Mode of birth			
*Vaginal birth*	756 (79)	102 (39)	*Reference*
*Vacuum extraction*	82 (9)	29 (11)	2.62 (1.64–4.20)
*Planned caesarean section*	88 (9)	33 (12)	2.78 (1.77–4.36)
*Emergency caesarean section*	27 (3)	100 (38)	27.5 (17.1–44)
Epidural anesthesia			
*Yes*	326 (35)	143 (55)	2.27 (1.72–3.01)
*No*	601 (65)	116 (45)	*Reference*
Postpartum variables			
Postpartum complications^e^			
*Yes*	124 (14)	111 (43)	4.70 (3.44–6.41)
*No*	771 (86)	147 (57)	*Reference*
Breastfeeding experience^e^			
*Negative*	45 (5)	64 (24)	6.45 (4.28–9.73)
*Positive*	907 (95)	200 (76)	*Reference*
Hands-on approach^f^			
*Yes*	141 (15)	104 (40)	3.76 (2.77–5.10)
*No*	811 (85)	159 (61)	*Reference*
Depressive symptoms at 6 weeks postpartum^g^			
*Yes*	121 (13)	42 (16)	1.31 (0.89–1.92)
*No*	819 (87)	217 (84)	*Reference*

^a^Odds Ratio and 95% Confidence Intervals

^b^Body Mass Index

^c^EPDS (Edinburgh Postnatal Depression Scale) ≥ 13

^d^Vaginal bleeding during pregnancy, significant Braxton-Hicks contractions, symphysiolysis, diabetes, hypothyroidism, hypertonia and preeclampsia

^e^Hemorrhage > 1000 ml, manual placental expulsion, Apgar score at 5 min > 7, admission to the neonatal unit and laceration grade III or IV

^f^During the first breastfeeding session

^g^EPDS≥12

Table 2 presents the distribution of the study variables by exclusive breastfeeding at six weeks postpartum and the univariate effects of associations. Background factors that were associated with increased odds for not exclusively breastfeeding at six weeks were age < 25 years, primiparity, low education, being unemployed and increasing BMI. On the other hand, women with a history of depression were less likely to breastfeed exclusively at six weeks postpartum. Regarding pregnancy variables, pregnancy complications, presence of depressive symptoms in pregnancy and caesarean section were also associated with not breastfeeding exclusively at six weeks postpartum. During the first breastfeeding session, a delayed initiation (> 2 h) of breastfeeding, a self-reported negative experience and experience of the hands-on approach were negative predictors of exclusive breastfeeding at six weeks postpartum. Noteworthy was the fact that women reporting depressive symptoms at six weeks postpartum were also more likely to report partial breastfeeding or cessation of breastfeeding.

**Table 2:** Distribution of study variables by exclusively breastfeeding or not at six weeks postpartum

	Exclusive breastfeeding at six weeks postpartum		
	Yes (*n* = 943)	No (*n* = 274)	
	*n* %	*n* %	OR (95% CI)^a^
Background variables			
Age (*years*)			
*< 25*	43 (4)	25 (9)	1.92 (1.09–3.36)
*25–34*	676 (72)	181 (66)	0.88 (0.64–1.21)
*≥ 35*	224 (24)	68 (25)	*Reference*
Parity			
*Primiparas*	431 (49)	146 (59)	1.51 (1.13–2.01)
*Multiparas*	449 (51)	101 (41)	*Reference*
Educational level			
*High school or lower*	153 (17)	77 (29)	2.06 (1.50–2.83)
*College/University*	767 (83)	187 (71)	*Reference*
Employment status			
*Unemployed*	62 (7)	35 (13)	2.13 (1.37–3.30)
*Employed/Student*	859 (93)	228 (87)	*Reference*
Pre-pregnancy BMI^b^ (*kg/m*^*2*^)			
*< 25*	683 (74)	161 (60)	*Reference*
*25–29*	178 (19)	72 (27)	1.72 (1.24–2.37)
*30–34*	50 (6)	18 (7)	1.53 (0.87–2.69)
*≥ 35*	12 (1)	15 (6)	5.30 (2.44–11.5)
History of depression			
*Yes*	252 (28)	104 (40)	1.72 (1.29–2.29)
*No*	655 (72)	157 (60)	*Reference*
Pregnancy and Birth variables			
Smoking at 17th week of pregnancy			
*Yes*	4 (0.5)	4 (1.7)	0.28 (0.07–1.13)
*No*	830 (99.5)	233 (98.3)	*Reference*
Depressive symptoms during pregnancy^c^			
*Yes*	105 (11)	49 (18)	1.74 (1.20–2.52)
*No*	838 (89)	225 (82)	*Reference*
Pregnancy complications^d^			
*Yes*	486 (52)	168 (62)	1.49 (1.13–1.97)
*No*	444 (48)	103 (38)	*Reference*
Mode of birth			
*Vaginal birth*	697 (74)	161 (59)	*Reference*
*Vacuum extraction*	86 (9)	25 (9)	1.26 (0.78–2.03)
*Planned caesarean section*	74 (8)	47 (17)	2.75 (1.84–4.12)
*Emergency caesarean section*	86 (9)	41 (15)	2.06 (1.37–3.11)
Epidural anesthesia			
*Yes*	359 (39)	110 (42)	1.12 (0.85–1.48)
*No*	563 (61)	154 (58)	*Reference*
Postpartum variables			
Postpartum complications^e^			
*Yes*	169 (19)	66 (26)	1.49 (1.07–2.06)
*No*	727,981)	191 (74)	*Reference*
Breastfeeding within 2 h after birth			
*No*	165 (18)	99 (36)	2.67 (1.98–3.59)
*Yes*	778 (82)	175 (64)	*Reference*
Breastfeeding experience			
*Negative*	57 (6)	52 (19)	3.64 (2.43–5.45)
*Positive*	885 (94)	222 (81)	*Reference*
Hands-on approach^f^			
*Yes*	145 (15)	100 (37)	3.16 (2.33–4.27)
*No*	796 (85)	174 (63)	*Reference*
Depressive symptoms at 6 weeks postpartum^g^			
*Yes*	111 (12)	52 (19)	1.78 (1.24–2.55)
*No*	820 (88)	216 (81)	*Reference*

^a^Odds Ratio and 95% Confidence Intervals

^b^Body Mass Index

^c^ EPDS (Edinburgh Postnatal Depression Scale) ≥ 13

^d^Vaginal bleeding during pregnancy, significant Braxton-Hicks contractions, symphysiolysis, diabetes, hypothyroidism, hypertonia and preeclampsia

^e^Hemorrhage > 1000 ml, manual placental expulsion, Apgar score at 5 min > 7, admission to the neonatal unit and laceration grade III or IV

^f^During the first breastfeeding session

^g^EPDS≥12

The multivariable logistic regression analysis for exclusive breastfeeding at six weeks postpartum is displayed in Table 3. According to the direct effect model (Model 1), both the presence of depressive symptoms during pregnancy (OR:1.93, 95% CI: 1.28–2.91) and not initiating breastfeeding within the first two hours after birth (OR: 2.61, 95% CI: 1.80–3.78) were independent significant predictors for not exclusively breastfeeding at six weeks postpartum. Among other variables included in the model, primiparas, women with lower educational level, and women giving birth by planned caesarean section compared to vaginal birth, were at increased odds of not exclusive breastfeeding at six weeks postpartum.

**Table 3:** Multivariable adjusted logistic regression results for not breastfeeding exclusively at six weeks postpartum

Variables	Model 1^a^	Model 2^b^
	OR (95% CI)^c^	OR (95% CI)
Mothers age when giving birth *(1-year increment)*	1.01 (0.98–1.05)	1.01 (0.97–1.05)
Parity (*Primiparas* vs. *Multiparas*)	1.44 (1.03–2.01)	1.10 (0.77–1.58)
Educational level (*High school or Lower* vs. *College/University*)	1.94 (1.34–2.81)	1.91 (1.31–2.79)
Mode of birth		
*Vaginal birth/Vacuum extraction*	Reference	Reference
*Planned caesarean section*	2.25 (1.42–3.55)	1.98 (1.24–3.18)
*Emergency caesarean section*	0.92 (0.55–1.54)	1.01 (0.59–1.72)
Breastfeeding within 2 h after birth *(No* vs. *Yes)*	2.61 (1.80–3.78)	1.96 (1.31–2.93)
Depressive symptoms during pregnancy^d^ *(Yes* vs. *No)*	1.93 (1.28–2.91)	1.70 (1.08–2.57)
History of depression *(Yes* vs. *No)*		1.47 (1.06–2.05)
Hands-on approach^e^ *(Yes* vs. *No)*		2.48 (1.71–3.59)
Breastfeeding experience *(Negative* vs. *Positive)*		1.70 (1.03–2.82)

^a^Model 1 corresponds to the direct effect model

^b^Model 2 corresponds to the indirect effect models, examining also the effect of mediator variables in the association of interest

^c^Odds Ratios and 95% Confidence Intervals

^d^EPDS≥13

^e^During the first breastfeeding session

The indirect effect model, investigating also the effect of the mediators in the odds of not exclusively breastfeeding at six weeks postpartum is also presented in Table 3 (Model 2). The association of depressive symptoms in pregnancy and initiation of breastfeeding later than six hours after birth, but also of lower education and planned caesarean section with not breastfeeding exclusively at six weeks postpartum remained in this model, but the effect of parity was lost. Additionally, the use of the hands-on approach in the first breastfeeding session, a negative first breastfeeding experience and a history of depression before pregnancy were associated with not exclusively breastfeeding at six weeks postpartum.

**Fig. 2:**
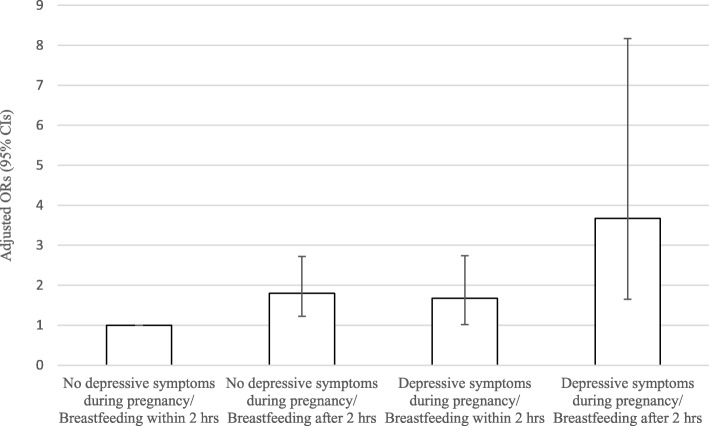
Multiple logistic regression analysis derived Odds Ratios (OR) and their 95% Confidence Intervals (95% CI) on the combined effect of depressive symptoms during pregnancy and breastfeeding or not within two hours after birth on the odds of exclusive breastfeeding at six weeks postpartum. The ORs are adjusted for mother’s age when giving birth, parity, educational level, mode of birth, history of depression, hands-on approach, and breastfeeding experience

When combining the two main exposure variables of interest (depressive symptoms during pregnancy and breastfeeding initiation within two hours after birth), as depicted in Fig. 2, women with depressive symptomatology who did not breastfeed within the first two hours after birth were at the highest risk for not breastfeeding exclusively at six weeks postpartum. Notably though, both variables, even on their own, significantly increased the odds. A power calculation based on our sample size showed that the sample was sufficiently powered (1-β > 80%) to detect as statistically significant (α = 0.05) a minimum OR of 1.59, as an association estimate between breastfeeding initiation later than two hours after birth and not exclusively breastfeeding at six weeks postpartum.

## Discussion

### Main findings

The current study identified a cumulative negative effect of the presence of depressive symptoms during pregnancy and a postponed first breastfeeding session on the duration of exclusive breastfeeding as assessed at six weeks postpartum. This indicates that women with depressive symptoms during pregnancy might be more vulnerable to the consequences of a postponed first breastfeeding session on breastfeeding later in the postpartum period.

### Strengths and limitations

Among the main strengths of this study are the large sample size and the number of studied variables on an individual level, which gave the possibility of controlling for multiple confounders in the analyses. On the negative side, it could be argued that women answered questions on breastfeeding initiation via self-report at six weeks postpartum, which poses an eventual problem of recall bias. Nevertheless, it has been shown that women a long time after giving birth, are capable of successfully recalling what happened during the birth process and the early hours postpartum [19]. Additionally, intention to breastfeed was not assessed in the questionnaires, as we assume that nearly all mothers in this Swedish setting had planned to breastfeed; indeed, all women included in the study actually initiated breastfeeding. Nevertheless, one could speculate that some women who delayed the breastfeeding initiation might have a lower commitment to breastfeeding, which could be reflected in not exclusively breastfeeding at six weeks postpartum. To be considered is also the fact that, women participating in the present study have a higher exclusive breastfeeding rate compared to the Swedish national and county-level average. Overall, the BASIC study has a participation rate of 22% pregnant women, probably due to extensive questionnaires and collection of biological material. The participating women have a slightly higher educational level than the background population in Uppsala and are slightly more often primiparas. These differences could affect the generalizability of the findings but they are not expected to greatly affect effect estimates. Further, the prevalence of perinatal depression in the BASIC study is very close to that of other studies, hence the lack of an attrition analysis should not introduce significant bias in our analyses. Lastly, the Edinburgh Postnatal Depression Scale, although widely recognized in medical research and also translated and validated in Swedish, [17] only detects depressive symptoms and does not provide a diagnosis of clinical depression.

### Interpretation

In our sample, one fifth of the mother-infant dyads did not have the opportunity to accomplish the first breastfeeding session within two hours after birth, despite the recommendations to initiate breastfeeding during the newborns’ initial alert period during the first hours after birth. This could be due to separation of mother and newborn after a caesarean section or another complication. At the Uppsala University Hospital, all infants being born by caesarean section are to be removed from the operating theatre and separated from the mother, for a short time period. If a midwife or nurse is not available to take responsibility for the infant in the recovery ward, the infant will continue to be separated from the mother and thus the first breastfeeding session will be delayed. Women experiencing larger vaginal tears or retained placenta might also be subjected to the same clinical routine. In the univariate analyses, delayed breastfeeding (> 2 h) after birth was also associated with primiparity and the use of epidural anesthesia during labour. Given that primiparas tend to also breastfeed for a shorter period compared to multiparas [20], women with no previous breastfeeding experience might be in greater need for targeted breastfeeding support. Regarding the use of anesthetics during labour, it has been suggested to affect the newborns reflexes, making it more problematic for them to latch on, and therefore possibly complicating and postponing the first breastfeeding session [21]. Further, we noted an increased frequency of the hands-on approach when the first breastfeeding session takes place more than two hours postpartum, as has been previously described [22]. The higher rate of the hands-on approach among those women could indicate a wish to help and support mother-infant dyads to establish breastfeeding, but this model of breastfeeding support has been shown to increase negative experience of breastfeeding among women [22, 23]. Accordingly, women with a postponed breastfeeding initiation, were more likely to reported a negative first breastfeeding experience.

Despite the recommendations on exclusive breastfeeding during the first 6 months, only 77% of the participating women were breastfeeding exclusively at 6 weeks postpartum. Potential risk factors for an early discontinuation of breastfeeding or partial breastfeeding at 6 weeks postpartum were lower age, primiparity, low maternal education, obesity and depressive symptoms during pregnancy which have all been investigated and pointed out in previous research [12, 13, 20, 24, 25]. Likewise, caesarean section and other obstetric complications during labour, as well as a postponed first breastfeeding session were associated with not exclusively breastfeeding at six weeks postpartum. Women undergoing a caesarean section might experience more pain postpartum affecting breastfeeding negatively [26]. Also, women undergoing a planned caesarean section for psychosocial reasons, such as for example fear of childbirth, might be more vulnerable, possibly also having a lower breastfeeding self-efficacy [27]. Further, our results indicate that a negative first breastfeeding session and the use of the hands-on approach were associated with exclusive breastfeeding lasting less than six weeks, which is also likely linked to a low breastfeeding self-efficacy.

As shown in earlier research [12, 13, 15], women breastfeeding exclusively at six weeks had lower odds for significant depressive symptoms than women who did not. Breastfeeding seems to be associated with decreased odds of postpartum depression, whereas early breastfeeding cessation or negative early breastfeeding experience, as indicated by breastfeeding aversion or severe breastfeeding pain have been associated with a higher risk [28, 29]. This potentially protective effect of breastfeeding against depressive symptoms has been suggested to be exerted via attenuation of the cortisol response to stress, oxytocin release, improvement of the mother’s breastfeeding self-efficacy, emotional involvement and interaction with the infant [13]. Conversely, mothers with a history of depression or those experiencing postpartum depression also more often report shorter breastfeeding duration [29, 30].

Therefore, this study adds to the available evidence investigating the cumulative effect of antenatal depression and that postponed breastfeeding initiation, which also has a negative impact on breastfeeding duration [6–8]. In our study, parallelly, depression during pregnancy and late initiation of breastfeeding interacted in increasing the odds for non-exclusive breastfeeding at six weeks postpartum. Our results show that at six weeks postpartum, women with depressive symptoms during pregnancy and breastfeeding for the first time later than two hours postpartum had almost a 4-fold increase in the odds of not breastfeeding exclusively at six weeks postpartum. Women with depressive symptoms during pregnancy are a vulnerable group, and previous studies have concluded that these women tend to breastfeed for a shorter period [29, 30]. Depressive symptoms as well as the timing of the first breastfeeding session are also linked to a lower breastfeeding self-efficacy, pointing to that these women are in need of targeted encouraging breastfeeding support to enhance the chances of a longer exclusive breastfeeding duration [9, 31].

## Conclusions

Our results show that women experiencing depressive symptoms during pregnancy, as well as those with a postponed first breastfeeding session, are more likely to not exclusively breastfeed at 6 weeks postpartum, which is the current recommendation. This could indicate a potential window of opportunity for intervention among the high-risk group of women with depressive symptoms in pregnancy within the first hours after birth, as they could possibly benefit from targeted breastfeeding support, thus enhancing the possibilities of longer exclusive breastfeeding duration. Lastly, if results from the current study could be confirmed in other settings, they should be disseminated among health care professionals in order to possibly revise routines during the first hours postpartum aiming to offer all women the possibility for early breastfeeding initiation of and subsequent adequate breastfeeding support, where needed.

## Additional file

Additional file 1: **Figure S1.** Graphical representation as directed acycled graph (DAG) of the conceptual model designed as to determine mediators and confounders in the association of interest between breastfeeding initiation at the first 2 h and exclusive breastfeeding at 6 weeks postpartum. (DOCX 334 kb)

## Abbreviations

BMI: Body Mass Index
CI: Confidence Intervals
DAG: Directly Acycled Graph
EPDS: Edinburgh Postnatal Depression Scale
OR: Odds ratio
SD: Standard Deviation
WHO: World Health Organization
