# Supplementary figures and images for: Antenatal depressive symptoms and early initiation of breastfeeding in association with exclusive breastfeeding six weeks postpartum: a longitudinal population-based study
Source: BMC Pregnancy Childbirth. 2019 Jan 29;19:49. doi: 10.1186/s12884-019-2195-9 (PMC6352434; doi:10.1186/s12884-019-2195-9)

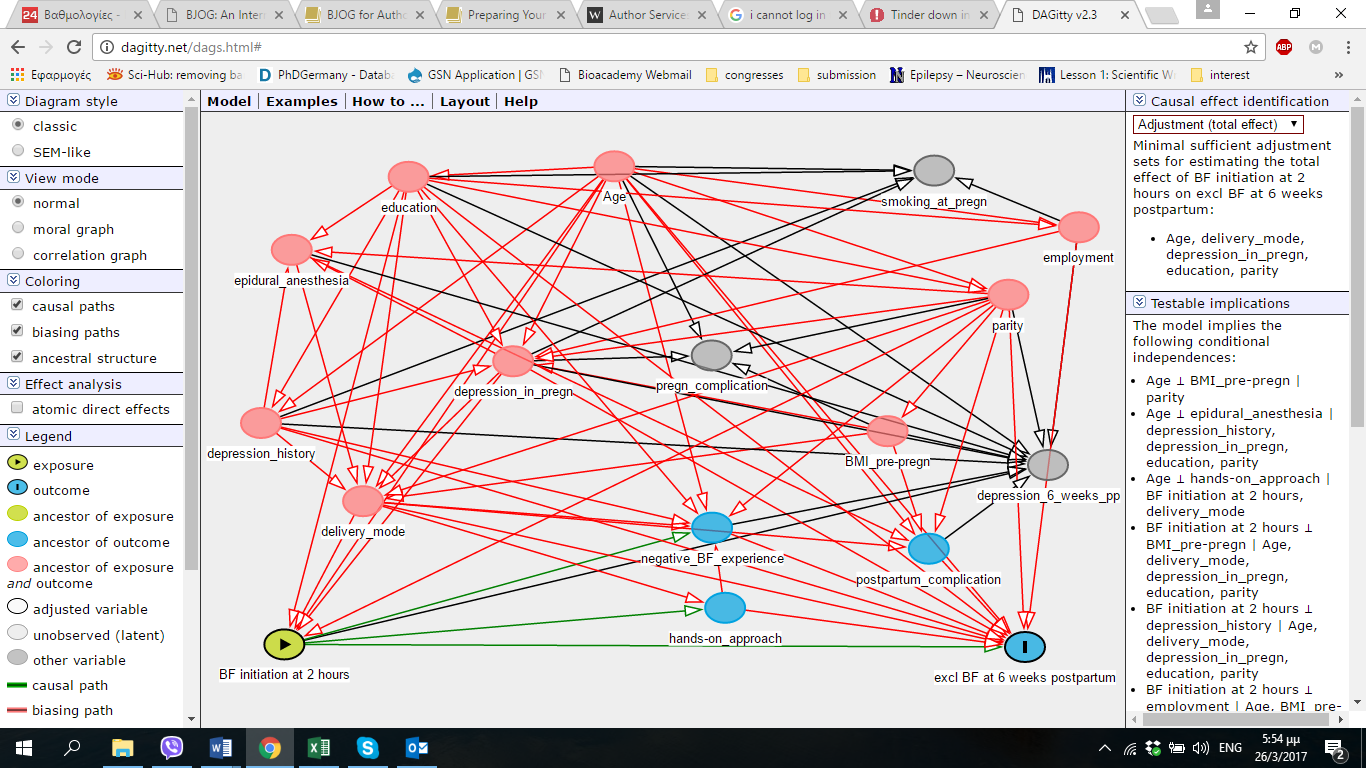

Supplement: Supplementary file 1 — Figure S1. Graphical representation as directed acycled graph (DAG) of the conceptual model designed as to determine mediators and confounders in the association of interest between breastfeeding initiation at the first 2 h and exclusive breastfeeding at 6 weeks postpartum. (DOCX 334 kb) [file 12884_2019_2195_MOESM1_ESM.docx]
